# Impact of differences in computed tomography value-electron density/physical density conversion tables on calculate dose in low-density areas

**DOI:** 10.1007/s13246-025-01611-4

**Published:** 2025-07-23

**Authors:** Mia Nomura, Shunsuke Goto, Mizuki Yoshioka, Yuiko Kato, Ayaka Tsunoda, Kunio Nishioka, Yoshinori Tanabe

**Affiliations:** 1 Faculty of Health Sciences, Department of Radiological Technology, Okayama University Medical School, Okayama University, 2-5-1 Shikata, Kita-ku, Okayama, 700-8558 Japan; 2 Graduate School of Health Sciences, Department of Radiological Technology, Okayama University, 2-5-1, Shikata, Kita, Okayama, 700-8525 Japan; 3 Department of Radiology, Tokuyama Central Hospital, 1-1 Kodacho, Shunan, Yamaguchi 745-8522 Japan; 4 Faculty of Medicine, Graduate School of Health Sciences, Okayama University, 2-5-1, Shikata, Kita, Okayama, 700-8525 Japan

**Keywords:** Computed tomography, Dose calculation, Inter-facility variation, Low-density regions, Percentage depth dose, Radiation therapy planning system

## Abstract

In radiotherapy treatment planning, the extrapolation of computed tomography (CT) values for low-density areas without known materials may differ between CT scanners, resulting in different calculated doses. We evaluated the differences in the percentage depth dose (PDD) calculated using eight CT scanners. Heterogeneous virtual phantoms were created using LN-300 lung and − 900 HU. For the two types of virtual phantoms, the PDD on the central axis was calculated using five energies, two irradiation field sizes, and two calculation algorithms (the anisotropic analytical algorithm and Acuros XB). For the LN-300 lung, the maximum CT value difference between the eight CT scanners was 51 HU for an electron density (ED) of 0.29 and 8.8 HU for an extrapolated ED of 0.05. The LN-300 lung CT values showed little variation in the CT-ED/physical density data among CT scanners. The difference in the point depth for the PDD in the LN-300 lung between the CT scanners was < 0.5% for all energies and calculation algorithms. Using Acuros XB, the PDD at − 900 HU had a maximum difference between facilities of > 5%, and the dose difference corresponding to an LN-300 lung CT value difference of > 20 HU was > 1% at a field size of 2 × 2 cm^2^. The study findings suggest that the calculated dose of low-density regions without known materials in the CT-ED conversion table introduces a risk of dose differences between facilities because of the calibration of the CT values, even when the same CT-ED phantom radiation treatment planning and treatment devices are used.

## Introduction

Radiation therapy is widely used to treat lung, prostate, liver, metastatic brain, and other cancers [1, 2]. The high precision of methods such as intensity-modulated radiation therapy, stereotactic radiation therapy, and stereotactic body radiation therapy (SBRT) allows localized lesions to receive high-dose radiation from multiple directions [3, 4]. SBRT is widely used to treat primary lung cancers with a tumor diameter of approximately 5 cm and metastatic lung cancers with few other lesions [3, 5].

SBRT for lung cancer typically delivers 50–60 Gy in 3–5 fractions, employing small radiation fields (~ 5 cm in diameter) and focusing on minimizing damage to adjacent organs. The presence of at-risk organs in the vicinity means that the prescribed dose uncertainty must be minimized [5, 6]. The uncertainty in SBRT for lung cancer is caused by various factors, such as the physical properties of the tumor, calculation accuracy of the algorithm, geometric accuracy of the collimator jaws or multileaf collimator of the radiation therapy device, and setup accuracy during the treatment period [7–9]. Improving the consistency of radiation therapy is essential for reducing dose uncertainty [10, 11].

The accuracy of dose calculations in radiation therapy planning systems (RTPS) for small radiation fields is affected by the unique physical characteristics of these fields and the inherent uncertainties associated with low-density tissues, such as the lungs [12, 13]. One significant uncertainty arises from the loss of lateral charged particle equilibrium (LCPE), which alters radiation quality and can decrease RTPS dose calculation accuracy [12–14]. Various calculation algorithms, including the superposition method and Monte Carlo simulation, are commonly employed in RTPS for lung cancer SBRT [14]. However, these methods exhibit differences in accuracy, making the selection of an appropriate algorithm crucial for delivering the prescribed dose effectively.

The dose calculation algorithms of the Eclipse (varian medical systems) treatment planning system include the analytical anisotropic algorithm (AAA) and Acuros XB (AXB). The AAA models have three separate contributions: primary photons, contaminating photons, and electrons. Each is characterized by a fluence, energy deposition function, and scatter kernel [15, 16]. The scatter kernel undergoes anisotropic scaling based on electron density (ED); AAA tends to reduce the calculation accuracy in sharp dose perturbations caused by air and high-density objects [15, 16]. In contrast, the dose calculation algorithms of AXB employ a linear Boltzmann transport equation solver to analyze the macroscopic behavior of radiation particles, including photons and electrons, as they interact with the material, calculating the dose based on the mass density and material type. The AXB achieves high accuracy in dose calculations in the presence of heterogeneities [14, 17].

In lung SBRT, a large dose can require a long irradiation time, which may result in the patient changing position during treatment or experiencing breathing disorders, leading to an intrafractional baseline shift or a drift in tumor position. Flattening filter-free (FFF) lung SBRT is useful because the dose rate can be increased [18]. However, FFF radiation treatment is likely to include low-energy radiation components because radiation does not pass through the flattening filter, resulting in changes in radiation quality [19]. Accurate treatment calculations for these changes in radiation quality require a precise understanding of the physical and ED of human tissues.

The RTPS calculates the dose distribution by considering the effect of tissue inhomogeneities, as determined by computed tomography (CT) and calibration of the Hounsfield units (HU) and relative ED/physical density (PD) curves [20]. The CT-ED/PD curve conversion table was generated using several materials with known EDs, such as the lungs, bones, and water; the relationship between the CT and ED/PD values enables the calculation of megavoltage (MV) treatment doses using kilovoltage CT images [17]. The HU value of the CT system was calibrated according to the manufacturer’s instructions, and the absolute CT values may differ between facilities. The International Electrotechnical Commission recommends that the mean CT value should not deviate by > 4 HU from the nominal values specified for the specific material of the test device [21, 22]. HU tolerances of ± 20 HU for soft tissue and ± 50 HU for lung and bone are recommended and would restrict dose changes in the treatment plan to < 1% [22, 23]. Using the CT-ED/PD curve, low-density CT values are calculated by interpolation and extrapolation in the absence of known materials [20, 24]. Therefore, dose calculations for these low-density areas may differ between facilities. The impact of low density on dose calculations may be greater for small radiation fields and FFF lung SBRT.

To the best of our knowledge, no study has evaluated interfacility differences in calculated doses for low-density areas without known materials in small-field FFF lung SBRT. In this study, we examined the doses calculated for low-density areas using CT-ED/PD curves from eight different facilities. We also determined the dose uncertainty in low-density areas in SBRT for lung cancer, where a known ED (PD) was not imaged. Minimizing this uncertainty in the future may improve the accuracy of radiation therapy calculations by developing low-density materials for CT-ED/PD tables.

## Methods

### Equipment

**Fig. 1:**
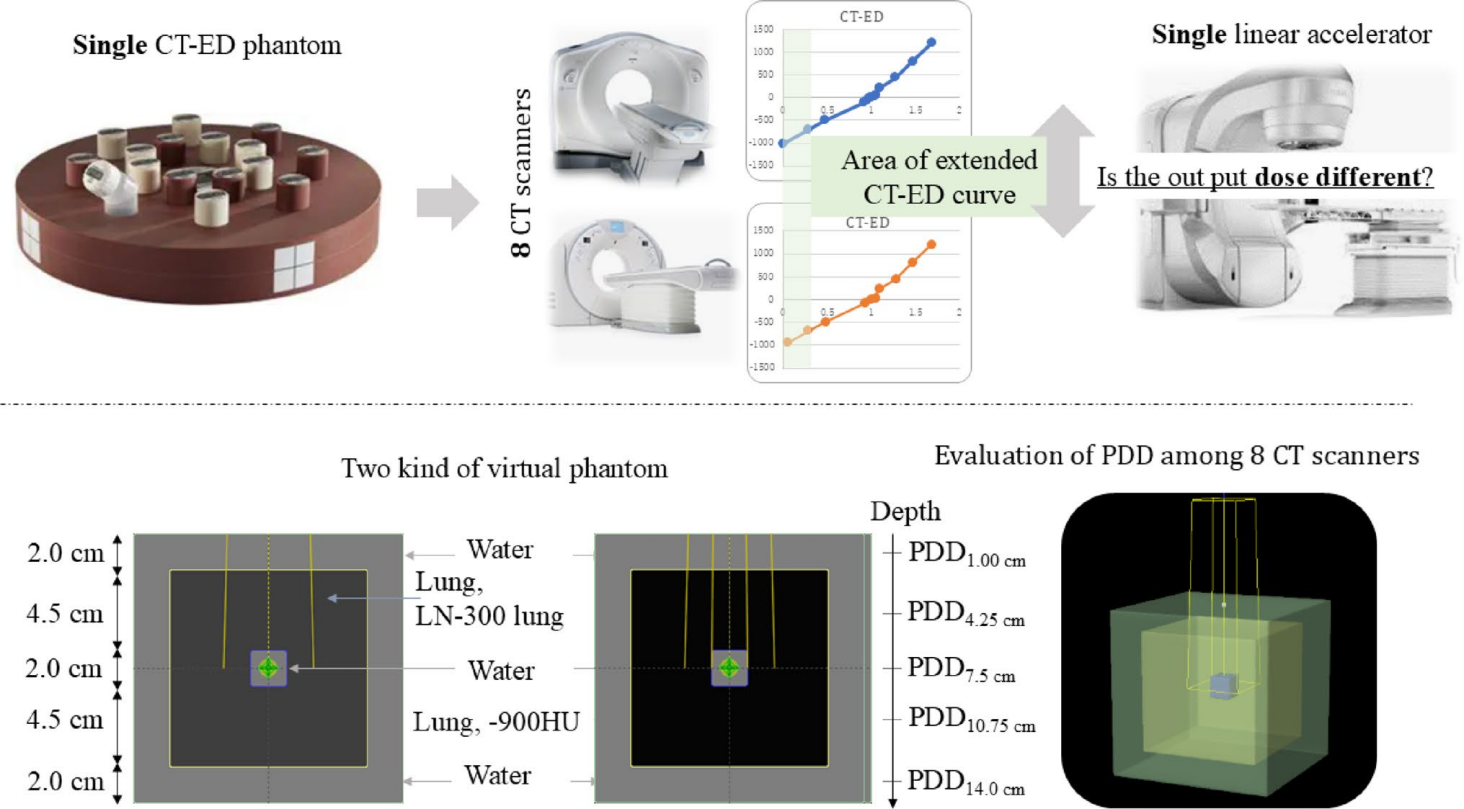
Flow diagram of the study. CT, computed tomography; ED, electron density; PDD, percentage depth dose

The eight CT scanners used included Lightspeed RT16 (General Electric, Boston, MA, USA), two Aquilion Exceed LB, Aquilion Prime SP, Aquilion Prime DE, two Aquilion Lightning, and Canon Aquilion Prime (Canon Medical Systems Corporation, Otawara, Japan). The CT-ED conversion curve was generated using a CT-ED phantom (Gammex Inc., Middleton, MI, USA), and the percentage depth dose (PDD) was calculated using Eclipse RTPS (version 16.01.00, Varian Medical Systems, Palo Alto, CA, USA). A flowchart of the study is presented in Fig. 1. We evaluated the impact of differences in the CT-ED/PD conversion table on PDD in low-density areas caused by variations in CT value calibration using inhomogeneous virtual phantoms. The virtual phantom was created based on a previous report that evaluated the accuracy of dose calculation algorithms for small fields in lung SBRT [12].

### Registration of CT-ED/PD table using eight CT scanners

The CT-ED phantom was scanned for nine materials (LN-300 lung, LN-450 lung, AP6 adipose, CT solid water, BRN-SR2 brain, inner bone, CB2-30% CaCO_3_a, CB2-50% CaCO_3_a, and SB3 cortical bone) using eight CT scanners at a tube voltage of 120 kVp. The CT values for air were set at − 1000 HU, corresponding to 0.0 electrons/gm, and the CT-ED/PD conversion curve was recorded in the RTPS (Fig. 1). For each ED, the average, minimum, maximum, and range of the CT values were computed (Table 1). As an example of low-density areas, a value of 0.05 electrons/gm from eight CT scanners was interpolated based on CT values between the LN-300 lung and air (Table 1).

**Table 1:** Relationship between electron density and CT value at eight CT scanners

Material	ED	Mean (HU)	Standard deviation	Min (HU)	Max (HU)	Max–min: difference (HU)
Interpolation value	0.05	− 950.5	3.2	− 955.2	− 946.4	8.8
LN-300 Lung	0.29	− 712.9	17.7	− 740.0	− 689.0	51.0
LN-450 Lung	0.48	− 509.6	9.5	− 518.2	− 491.6	26.6
AP6 Adipose	0.94	− 73.2	3.5	− 79.8	− 62.8	17.0
CT Solid Water	1.00	− 0.00	5.7	− 7.9	9.04	17.0
BRN-SR2 Brain	1.05	33.2	8.2	21.8	71.1	49.3
Inner Bone	1.10	221.3	9.2	179.3	241.8	62.5
CB2-30% CaCO_3_a	1.28	460. 4	15.9	440.0	496.2	56.2
CB2-50% CaCO_3_a	1.47	832.6	20.7	804.3	889.7	85.5
SB3 cortical bone	1.69	1,259.4	30.7	1,201.3	1,326.6	125.2

### Calculation of PDD curve for inhomogeneous virtual phantom by changing CT value for lung material

The heterogeneous virtual phantom was created using two types of three-layered cubic phantoms (outermost: water, 15 cm^3^; inner: lung, 9 cm^3^; center: water, 2 cm^3^) with an altered LN-300 lung of the CT value or -900 HU for lung material using the RTPS (Figs. 1). For the two types of virtual phantoms, the PDD on the central axis was calculated using five energies (4 MV, 6 MV, 10 MV, 6 MV FFF, and 10 MV FFF), irradiation field sizes of 2 × 2 cm^2^ and 5 × 5 cm^2^, two types of calculation algorithms (AAA and AXB), a source surface distance of 100 cm, and 100 monitor units.

### Comparative evaluation of the PDD of inhomogeneous virtual phantoms

The PDD and difference in PDD for the two types of inhomogeneous virtual phantoms were compared using two calculation algorithms and two field sizes. The difference in PDD was defined as the difference between the eight CT scanner PDDs and the average PDD of all the CT scanners. The differences in PDDs at five depths (PDD_1.00 cm_, PDD_4.25 cm_, PDD_7.5 cm_, PDD_10.75 cm_, and PDD_14.0 cm_) of the center of the water and lung in the virtual phantom were compared for both field sizes and calculation algorithms (Fig. 1), and both the average and absolute maximum values of the eight CT scanners were calculated for the two calculation algorithms and two field sizes.

### Evaluation of depths of maximum difference in PDD

The depth of the maximum difference in the PDD for the two types of inhomogeneous virtual phantoms was investigated using two calculation algorithms and two field sizes. The dose distribution for highly heterogeneous regions of tissue interfaces was evaluated with a difference in the PDD value from 5.8 cm to 6.8 cm depth for two irradiation field sizes in inhomogeneous virtual phantoms with -900 HU.

## Results

**Fig. 2:**
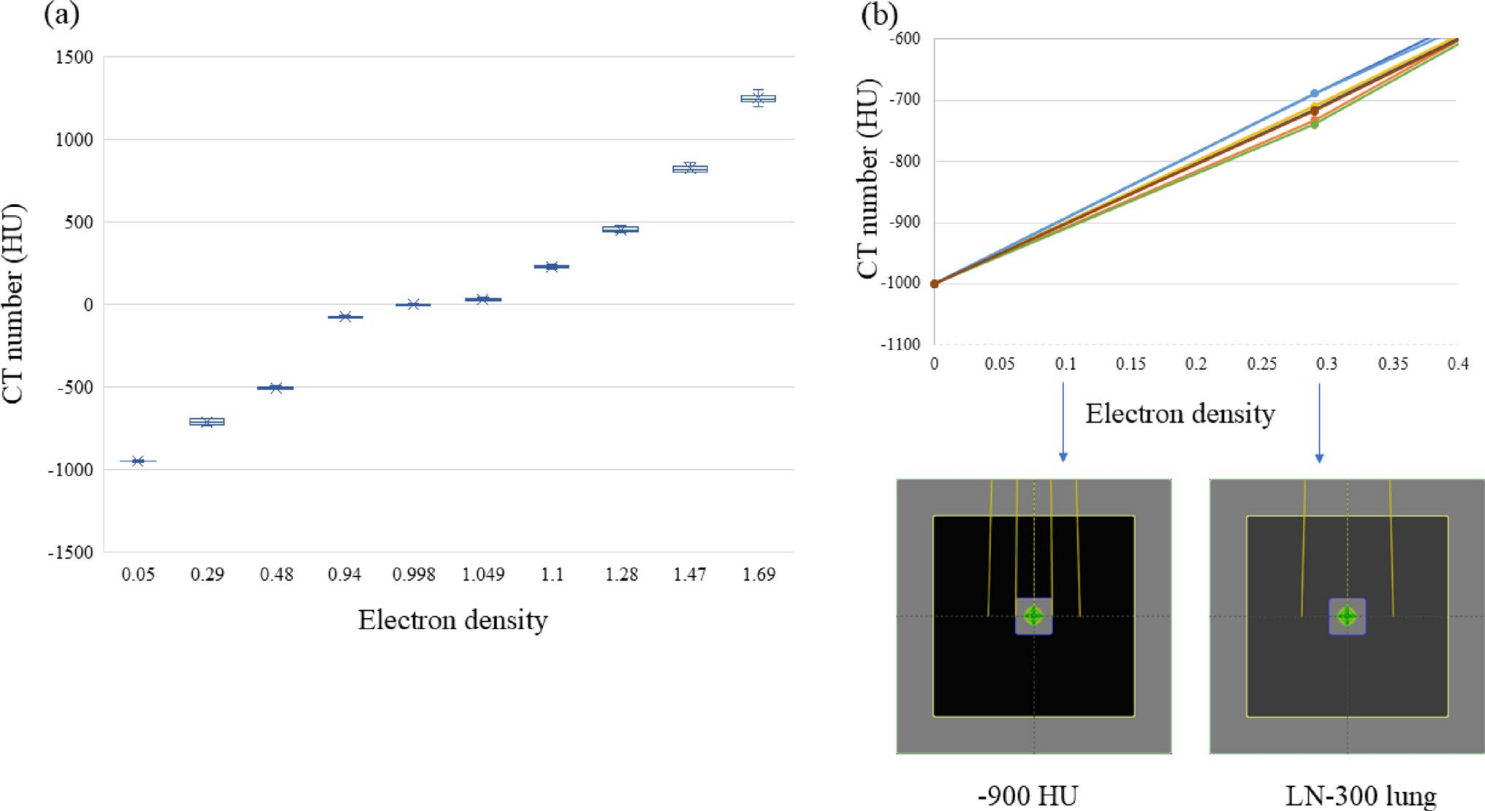
Relationship between electron density and CT value across eight computed tomography (CT) scanners. The CT value-to-electron density conversion curves for the eight CT scanners are shown in **a** all areas and **b** low-density areas using two types of inhomogeneous virtual phantoms

Table 1 and Fig. 2 illustrate the relationship between ED and CT values for the eight CT scanners evaluated. For the LN-300 lung phantom, the maximum observed difference in CT values across the scanners was 51 HU at an ED of 0.29 and 8.8 HU at an ED of 0.05. The CT value at 0.05 ED was interpolated between the values for the LN-300 lung and air.

**Fig. 3:**
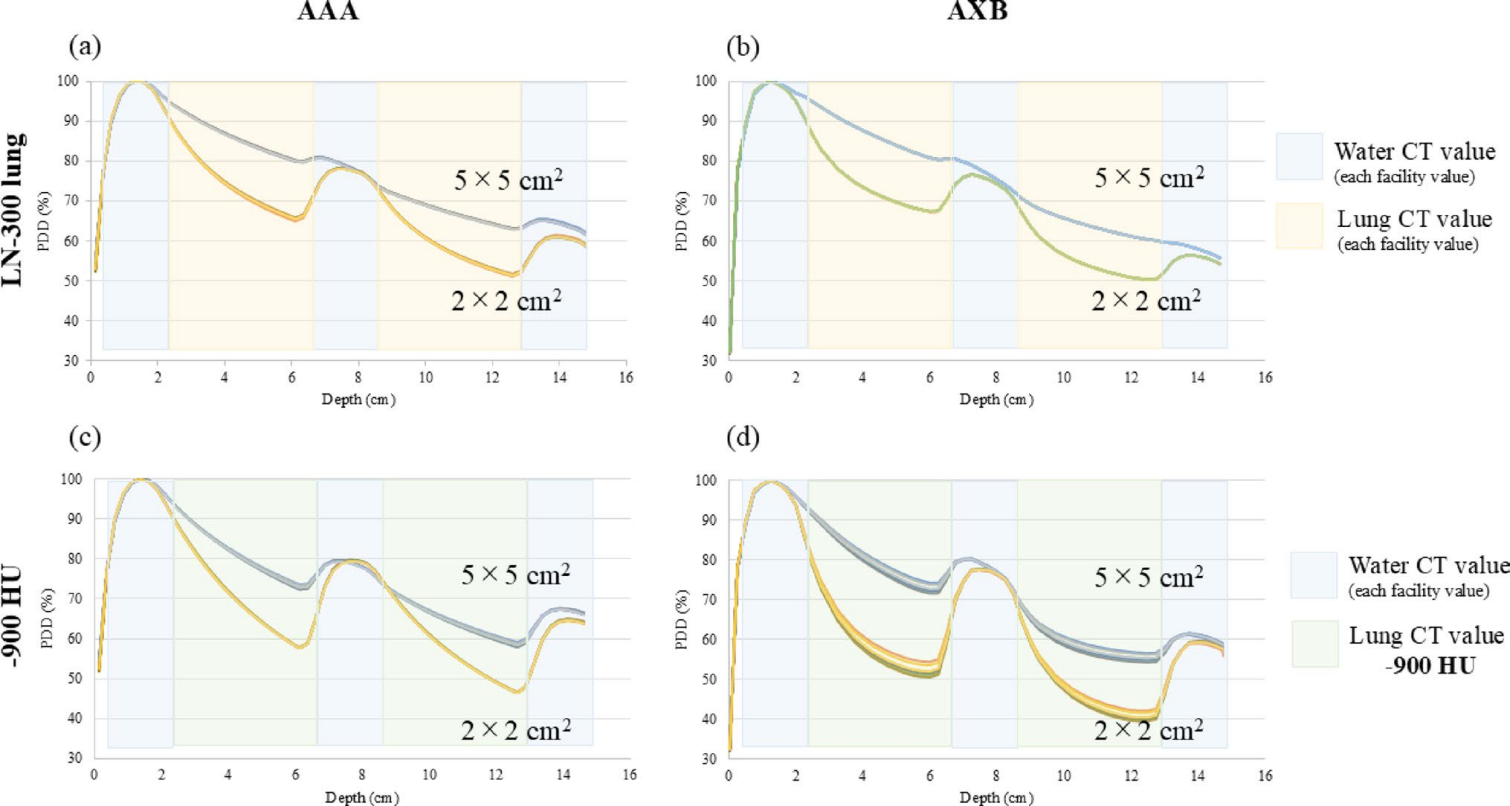
Relationship between changes in PDDs of eight CT scanners, with and without known CT values, for the lung component of a 6 MV virtual phantom. The PDD of the LN-300 lung using **a** the AAA algorithm or **b** the AXB algorithm. The PDD at − 900 HU using **c** the AAA algorithm or **d** the AXB algorithm. AAA, anisotropic analytical algorithm; AXB, Acuros XB; CT, computed tomography; HU, Hounsfield unit; PDD, percentage depth dose

**Fig. 4:**
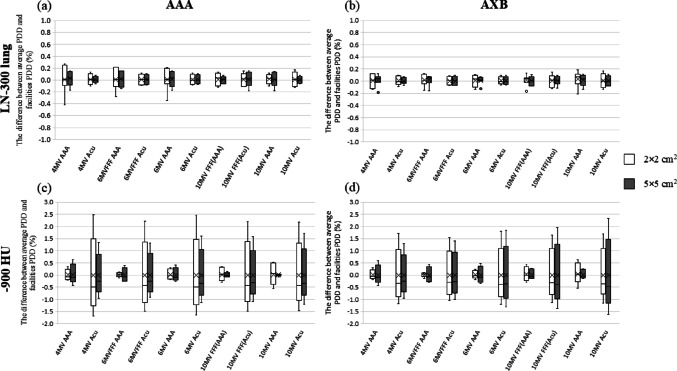
Results of the differences in PDD for each calculation algorithm and depth. The difference in PDD of the LN-300 lung at **a** PDD_4.25_ cm or **b** PDD_10.75_, and at **− **900 HU at **c** PDD_4.25_ cm or **d** PDD_10.75 cm_. AAA, anisotropic analytical algorithm; AXB, Acuros XB; CT, computed tomography; FFF, flattening filter-free; HU, Hounsfield unit; MV, megavolt; PDD, percentage depth dose

**Fig. 5:**
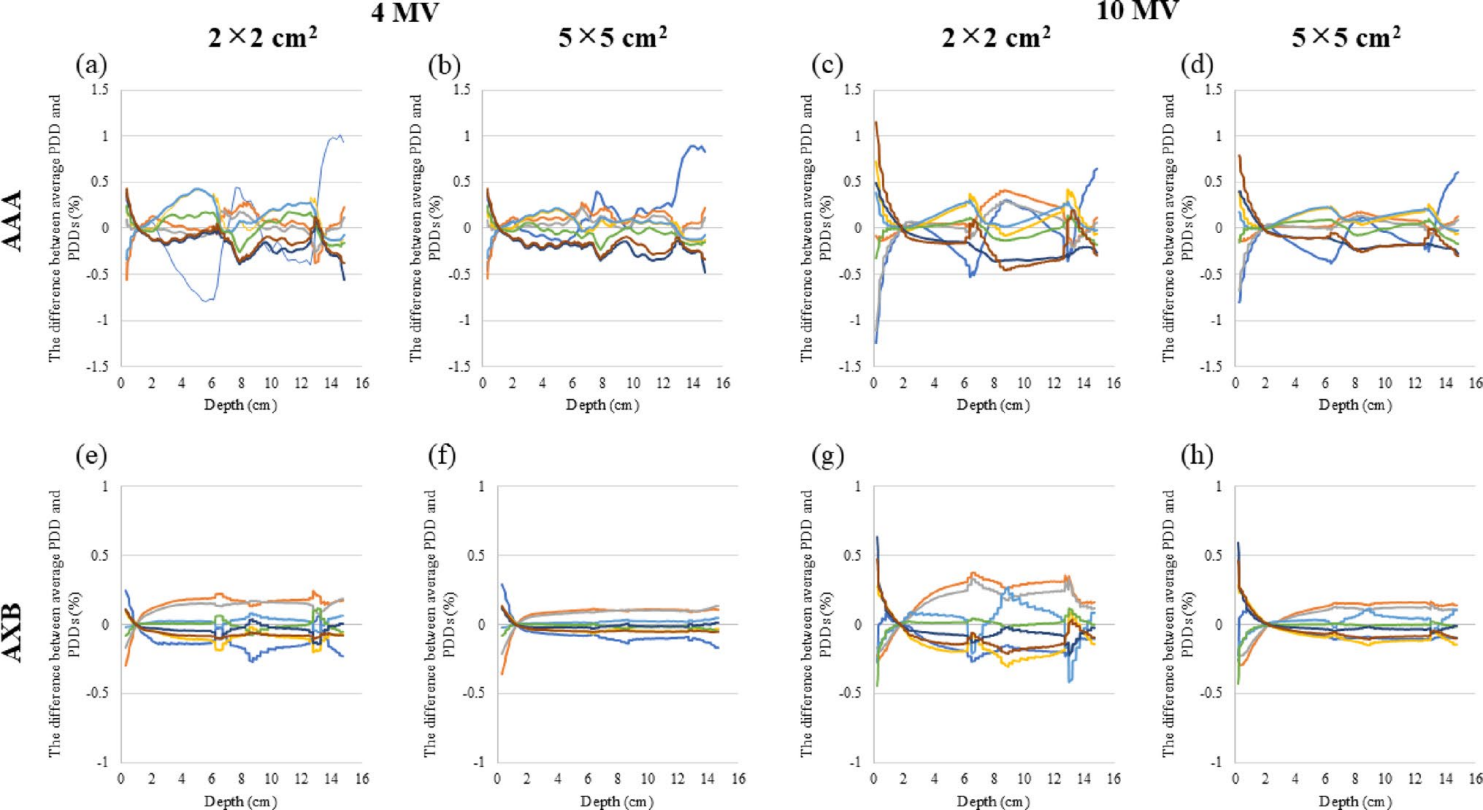
Deviation of PDDs of eight CT's from the average PDD when the low-density area is assigned to the CT value of LN-300 lung, using the following parameters: **a** 4 MV AAA field size 2 × 2 cm^2^, **b** 4 MV AAA field size 5 × 5 cm^2^, **c** 10 MV AAA field size 2 × 2 cm^2^, **d** 10 MV AAA field size 5 × 5 cm^2^, **e** 4 MV AXB field size 2 × 2 cm^2^, **f** 4 MV AXB field size 5 × 5 cm^2^, **g** 10 MV AXB field size 2 × 2 cm^2^, and **h** 10 MV AXB field size 5 × 5 cm^2^. AAA, anisotropic analytical algorithm; AXB, Acuros XB; CT, computed tomography; MV, megavolt; PDD, percentage depth dose

Figure 3 illustrates the PDDs of 6 MV for the two types of inhomogeneous virtual phantoms in the LN-300 lung or − 900 HU. The variance in the PDDs between the CT scanners in the LN-300 was minor compared to that of—900 HU. At − 900 HU, the difference in the PDDs of AAA was negligible compared to that of AXB, and the discrepancy in radiation fields of 5 × 5 cm^2^ was minimal compared to that of 2 × 2 cm^2^. Figure 4 and Table 2 display the differences in the PDD for each calculation algorithm and depth. In the inhomogeneous virtual phantoms of the LN-300 lung, the difference in PDD between the CT scanners was < 0.5% for all energies and calculation algorithms. At − 900 HU, the difference in PDD between the CT scanners was < 1.0% for AAA and < 2.5% for AXB across all energies tested. Moreover, at − 900 HU using AXB, the difference was more pronounced with a field size of 2 × 2 cm^2^ at a depth of 4.25 cm, whereas at higher energies, the variance was greater with a field size of 5 × 5 cm^2^ at a depth of 10.75 cm. The discrepancies between the average and eight CT scanner PDDs are shown in Fig. 5. The variation in the PDDs of the LN-300 lung was within 1% at all depths and was slightly greater for the 2 × 2 cm^2^ field size than for the 5 × 5 cm^2^ field size and for AAA than for AXB (Fig. 5).

**Table 2:** Difference in PDD for each calculation algorithm and each depth

Difference in PDD (%)		Algorithm	Lung, LN-300 lung					Lung, CT value -900HU				
			4MV	6MV	10MV	6MV FFF	10MV FFF	4MV	6MV	10MV	6MV FFF	10MV FFF
2 × 2	PDD_1.00_	AAA	0.1	0.2	0.5	0.1	0.2	0.2	0.2	0.2	0.1	0.2
		AXB	0.0	0.1	0.2	0.1	0.2	0.0	0.1	0.2	0.1	0.1
	PDD_4.25_	AAA	0.7	0.6	0.2	0.5	0.2	0.6	0.5	1.1	0.2	0.7
		AXB	0.2	0.2	0.3	0.2	0.3	4.2	4.1	3.6	3.7	3.7
	PDD_7.50_	AAA	0.5	0.3	0.5	0.4	0.4	0.6	0.6	0.8	0.6	0.7
		AXB	0.3	0.2	0.5	0.2	0.4	0.5	0.3	0.7	0.4	0.5
	PDD_10.75_	AAA	0.3	0.2	0.4	0.3	0.3	0.5	0.4	1.2	0.3	0.7
		AXB	0.2	0.2	0.3	0.2	0.3	2.9	3.0	2.8	2.6	2.8
	PDD_14.00_	AAA	0.7	0.6	0.4	0.6	0.5	0.7	0.6	0.7	0.6	0.7
		AXB	0.2	0.1	0.2	0.1	0.1	0.7	0.4	0.3	0.5	0.1
5 × 5	PDD_1.00_	AAA	0.1	0.2	0.5	0.1	0.3	0.2	0.2	0.2	0.1	0.2
		AXB	0.1	0.1	0.3	0.1	0.3	0.1	0.1	0.2	0.1	0.3
	PDD_4.25_	AAA	0.3	0.3	0.3	0.3	0.3	1.1	0.7	0.1	0.6	0.2
		AXB	0.2	0.2	0.1	0.2	0.1	2.3	2.7	2.9	2.2	2.7
	PDD_7.5_	AAA	0.4	0.3	0.3	0.3	0.3	0.7	0.4	0.4	0.4	0.4
		AXB	0.2	0.2	0.2	0.2	0.2	0.4	0.4	0.4	0.4	0.4
	PDD_10.75_	AAA	0.3	0.2	0.3	0.2	0.2	1.0	0.8	0.4	0.7	0.4
		AXB	0.1	0.2	0.2	0.2	0.2	2.3	3.2	4.0	2.4	3.3
	PDD_14.00_	AAA	0.7	0.6	0.4	0.6	0.5	0.6	0.4	0.3	0.4	0.3
		AXB	0.1	0.2	0.2	0.1	0.2	0.6	0.5	0.4	0.5	0.2

**Fig. 6:**
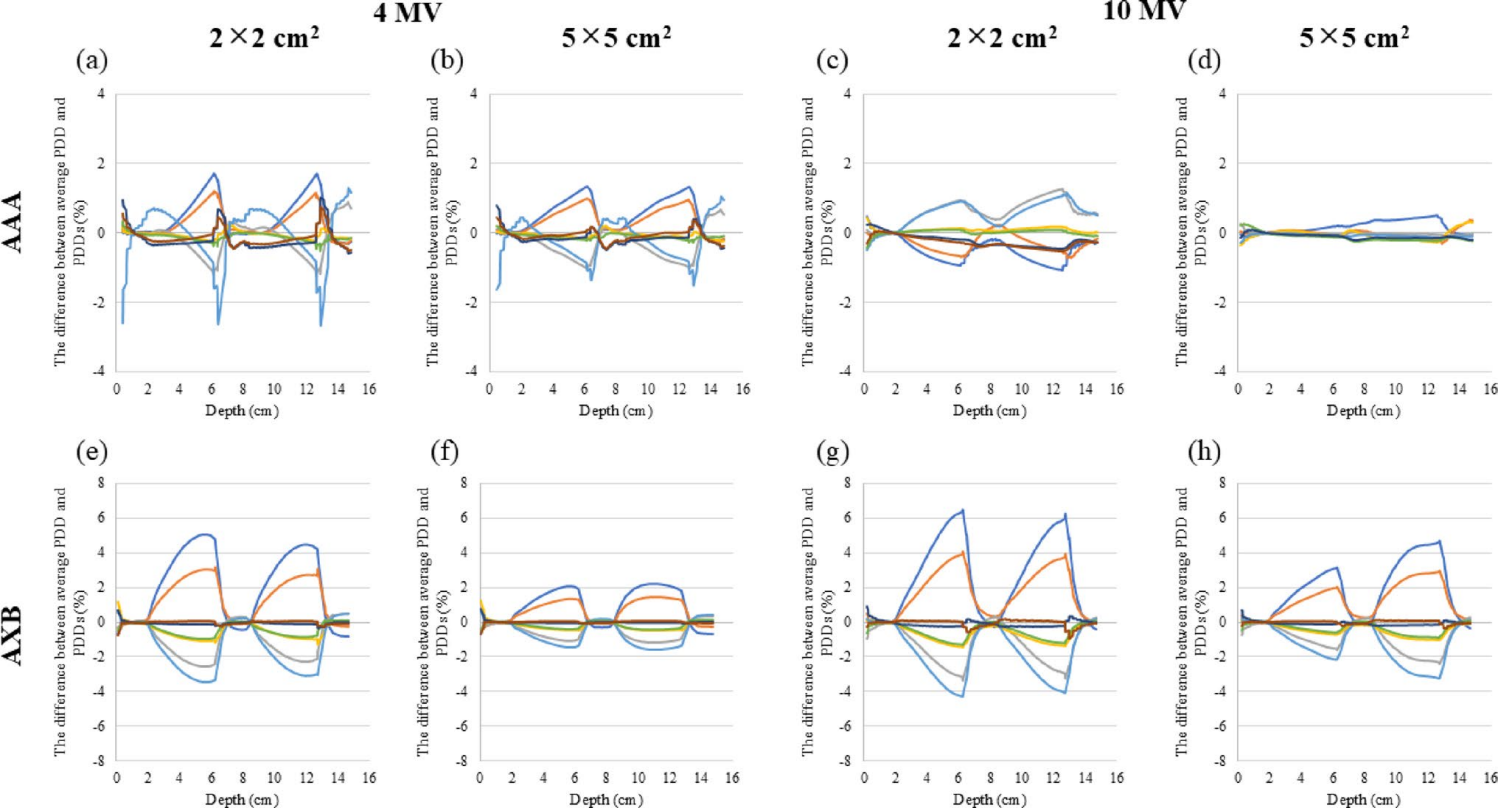
Deviation of the PDDs of eight CT scanners from the average PDD when the low-density area is assigned to the CT value -900 HU, using the following parameters: **a** 4 MV AAA, field size 2 × 2 cm^2^, **b** 4 MV AAA, field size 5 × 5 cm^2^, **c** 10 MV AAA, field size 2 × 2 cm^2^, **d** 10 MV AAA, field size 5 × 5 cm^2^, **e** 4 MV AXB, field size 2 × 2 cm^2^, **f** 4 MV AXB, field size 5 × 5 cm^2^, **g** 10 MV AXB, field size 2 × 2 cm^2^, and **h** 10 MV AXB, field size 5 × 5 cm^2^. AAA, anisotropic analytical algorithm; AXB, Acuros XB; CT, computed tomography; HU, Hounsfield unit; MV, megavolt; PDD, percentage depth dose

The PDD at -900 HU calculated using AXB indicated a maximum difference of > 10% among the eight CT scanners, and this difference from the average PDD was more pronounced for a field size of 2 × 2 cm^2^ than for a field size of 5 × 5 cm^2^ (Fig. 6). The difference in the PDD at -900 HU calculated with AAA generally increased as the energy decreased, whereas the difference calculated with AXB generally increased as the energy increased, with the deviation from the average PDD becoming larger at greater depths.

At -900 HU, the maximum dose difference between the CT scanners was larger when calculated with AXB than with AAA, as shown in Table 3. Using AXB, the dose difference was larger as the energy increased, whereas using AAA, the opposite trend was observed. The depth of the maximum dose difference between the CT scanners for both AAA and AXB increased as the energy increased at a field size of 5 × 5 cm^2^; little change occurred at a field size of 2 × 2 cm^2^.

**Table 3:** Maximum dose difference between CT scanners of CT value of Lung: − 900 HU

Max dose difference	Absolute average (max) (%)				Median depth(cm)			
	2 × 2		5 × 5		2 × 2		5 × 5	
	AAA	AXB	AAA	AXB	AAA	AXB	AAA	AXB
4MV	0.65 (1.58)	1.09 (2.69)	0.47 (1.00)	0.61 (1.55)	6.78	5.88	6.78	5.50
6MV FFF	0.22 (0.44)	0.93 (2.41)	0.27 (0.65)	0.64 (1.55)	7.38	6.25	6.75	6.25
6MV	0.18 (0.41)	1.14 (2.81)	0.32 (0.75)	0.79 (1.95)	10.36	6.25	6.25	6.13
10MVFFF	0.22 (0.54)	1.01 (2.51)	0.17 (0.38)	0.84 (2.04)	9.49	6.23	12.60	12.23
10MV	0.33 (0.55)	1.01 (2.75)	0.15 (0.37)	1.02 (2.54)	7.49	6.25	12.74	12.74

**Fig. 7:**
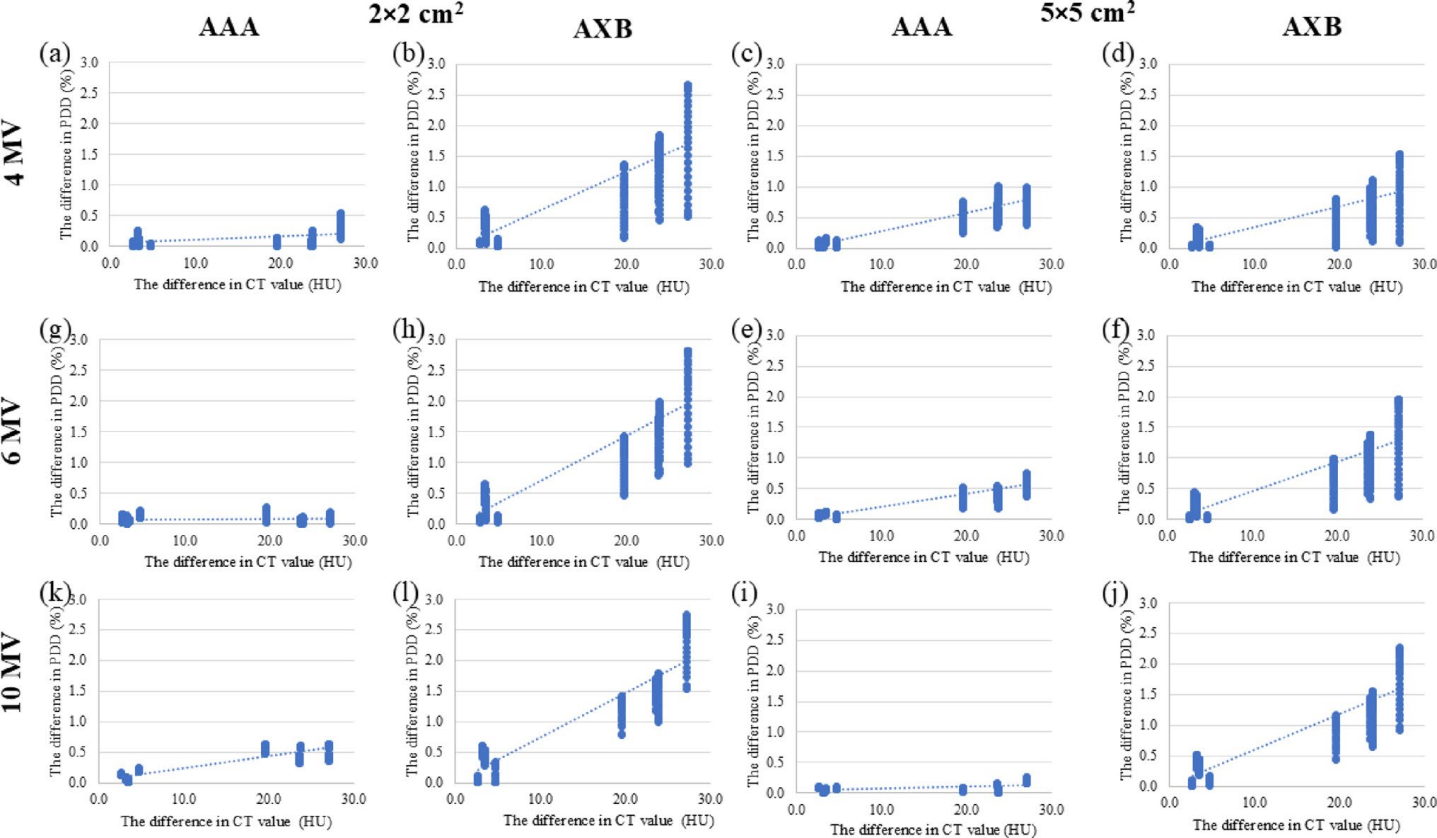
Relationship between the difference in PDD and the difference in CT value for the LN-300 lung at − 900 HU for PDD_5.8_–PDD_6.8_ at 4 MV. The straight lines represent linear approximation curves. **a** AAA, 2 × 2 cm^2^; **b** AXB, 2 × 2 cm^2^; **c** AAA, 5 × 5 cm^2^; **d** AXB, 5 × 5 cm^2^, 6 MV; **e** AAA, 2 × 2 cm^2^; **f** AXB, 2 × 2 cm^2^; **g** AAA, 5 × 5 cm^2^; **h** AXB, 5 × 5 cm^2^, 10 MV; **e** AAA, 2 × 2 cm^2^; **f** AXB, 2 × 2 cm^2^; **g** AAA, 5 × 5 cm^2^; and **h** AXB, 5 × 5 cm^2^. AAA, anisotropic analytical algorithm; AXB, Acuros XB; CT, computed tomography; HU, Hounsfield unit; MV, megavolt; PDD, percentage depth dose

Figure 7 depicts the relationship between the difference in PDD and the difference in CT values for the LN-300 lung at − 900 HU for PDD_5.8_–PDD_6.8_. Using AXB, the dose difference corresponding to a CT value difference of > 20 HU was > 1% at a field size of 2 × 2 cm^2^. Using the AAA, the dose difference corresponding to a CT value difference of > 30 HU was < 1% for all energies and two field sizes in the linear approximation equation.

## Discussion

This study evaluated the impact of the calculated dose in low-density areas by differences in CT-ED/PD conversion tables among eight CT scanners using the calculated PDD of two types of inhomogeneous virtual phantoms. We found large variations in the CT values of low-density materials, with LN-300 lung differing by up to 51 HU between the CT scanners. Furthermore, the calculated dose of low-density areas without known materials for AXB showed a large difference between the CT scanners and AAA. Previous reports have revealed that AAA has a lower calculation accuracy than AXB in low-density areas; however, differences between CT scanners have not yet been reported [23, 25]. This study suggests variations in the calculated dose of AXB by extrapolating the CT-ED curve, with large variations for small radiation fields due to the loss of LCPE. However, as there were no differences between the CT scanners when calculating the known materials of the CT-ED phantom components using AXB, we believe that an appropriate dose can be calculated by selecting an appropriate prescription point or dose-volume-based prescription [26, 27].

The change in the PDD between the LN-300 lung and -900 HU CT values was steeper at lower densities and smaller radiation field sizes. This is because low-density regions absorb less radiation, leading to increased forward scattering. Moreover, electrons traversing low-density regions travel greater distances, increasing the likelihood of losing LCPE, which reduces dose calculation accuracy [28]. In addition, although there was a slight variation between the CT scanners in the PDD of the LN-300 lung at -900 HU, there was a tendency for the difference to be greater using AXB. The AAA may not calculate slight differences in CT values owing to its limited ability to model electron-photon interactions across interfaces, including forward/backward scattered photons and the loss of LCPE [28, 29].

Evaluating PDD differences following the extension of the CT-ED curve at -900 HU showed that AXB had a large difference at a depth of 4.25 cm and a field size of 2 × 2 cm^2^, whereas at a depth of 10.75 cm, the difference between CT scanners was larger at a field size of 5 × 5 cm^2^ for higher energies. A small field size may affect the hardening of the photon energy spectrum, and partial LCPE may be established at greater depths even in small radiation fields, which may explain this difference [13].

Solid water was recorded as water in this study, showing a variance of approximately 17 HU across CT scanners. The use of the AAA may have resulted in a difference of approximately 0.5% in the PDD and could have induced fluctuations due to routine calibration [29]. The variations in the PDD at depths of 6–8 cm and 12–14 cm are likely attributable to the backscatter model in the rebuilding area, with minor differences depending on the CT value and energy. Furthermore, a discrepancy in the maximum dose was noted in the lung area adjacent to the rebuilding zone, and the median depth was shallower in the AXB region than in the AAA region, likely due to more intensive adjustments for backscattering where the lung tissue transitions to water [30]. The organs at risk of low density, such as the lung and rectal gas, are expected to have large variations for the isodose lines of lower dose and dose distribution for highly heterogeneous regions of tissue interfaces [24, 31].

For the ED and CT values of CT scanners, the registered ED value for the RTPS was as high as 0.29 for the LN-300 lung, and the minimum value of 0.0 corresponded to a CT value of -1000 HU across all CT scanners. Consequently, we deduce that variations in CT values of LN-300 lung across different CT machines introduce a dose uncertainty ranging from 0.29 to 0.00 electrons/gm in low-density areas. This study demonstrated that CT value discrepancies of LN-300 lung greater than 20 HU between CT scanners were associated with dose differences exceeding 1% in extrapolated low-density lung areas and nasal cavities with a field size of 2 × 2 cm^2^. Acknowledging the uncertainty present is critical, particularly in regions of highly heterogeneous tissue interfaces and dose distributions within small irradiation fields.

A limitation of this study was the use of a CT value for air set at -1000 HU without direct measurement of air CT HU. This may cause slight variations in the results compared to those obtained using air CT HU measured in the specific facility. It is important to note that air CT HU can vary depending on scan parameters and conditions [30, 32]. Further evaluations, including analysis of phantom production accuracy, tissue substitute choice, and CT scan conditions, could provide a more comprehensive assessment of the impact of the calculated dose using the CT-ED table [32]. In addition, the limitation of this study is that the evaluation was performed at the center; the off-axis change in the PDD could not be evaluated. As it has been reported that off-axis modeling accuracy differs considerably, caution is required when interpreting our findings. However, understanding the uncertainty of low-density regions on the central axis between CT scanners will aid in designing future treatment plans, which should avoid prescriptions of ultra-low-density regions. Radiotherapy for low-density areas, such as the head and neck, nasal cavity, lungs, and abdomen, is often treated; therefore, it is desirable to add known low-density materials to the CT-ED/PD table for accurate dose calculations in the future.

## Conclusion

The calculated dose of low-density regions without known materials in the CT-ED conversion table differed by up to 5% between CT scanners because of the calibration of the CT value even when the same CT-ED phantom, radiation treatment planning device, and treatment device were used. However, as the absorbed dose was low, future treatment plans can avoid prescriptions at the isocenter of ultralow-density regions and volume that extend into low-density areas, which will help address this issue.

## Data Availability

The data used to support the findings of this study are available from the corresponding authors upon request.
